# Pediatric Burns: A Systematic Review and Meta-Analysis on Epidemiology, Gender Distribution, Risk Factors, Management, and Outcomes in Emergency Departments

**DOI:** 10.7759/cureus.49012

**Published:** 2023-11-18

**Authors:** Jumanah Y Nassar, Abdullah A Al Qurashi, Ibrahim Abdullah Albalawi, Houriah Y Nukaly, Ibrahim R Halawani, Abdulaziz F Abumelha, Adnan M Osama Al Dwehji, Mahinar M Alhartani, Alanoud Asaad, Arwa Alnajashi, Imad M Khojah

**Affiliations:** 1 Medicine, Batterjee Medical College, Jeddah, SAU; 2 Medicine, King Saud bin Abdulaziz University for Health Sciences, Jeddah, SAU; 3 Medicine, Tabuk University for Health Science, Tabuk, SAU; 4 Medicine, King Abdulaziz University, Jeddah, SAU; 5 Medicine, King Saud bin Abdulaziz University for Health Sciences, Riyadh, SAU; 6 Medicine, Alfaisal University College of Medicine, Riyadh, SAU; 7 Emergency Medicine, King Abdulaziz University, Jeddah, SAU

**Keywords:** emergency room pediatric, pediatric, burn injury, plastic and reconstructive surgery, household risk factors, burn prevention, total body surface area (tbsa), scald injuries, emergency departments, pediatric burns

## Abstract

Pediatric burns pose a significant public health concern, ranking as the fifth most common nonfatal injury globally. This review consolidates data on the epidemiology, outcomes, and management of pediatric burns presenting to emergency departments. A systematic review was conducted across multiple databases, yielding 22 articles from 1992 to 2020. Utilizing the methodological index for non-randomized studies (MINORS) instrument, non-comparative studies scored from 2 to 11 with an average of 6.87, while comparative studies ranged from 12 to 16, averaging 13.67. The review included a total of 828,538 pediatric patients who were evaluated in the systematic review. Predominantly male victims ranged from 53% to 83%. The youngest victims were aged between 0 to 4 years. Burn etiology was largely attributed to scalds. A majority suffered from second-degree burns, with some studies reporting up to 89%. Limited data on total body surface area (TBSA) were documented, with only 2.5% requiring hospitalization. Common interventions included immediate resuscitation and skin grafting. Essential areas for future research are identified, including household risks, pre-treatment decisions, and the significant role of family dynamics in burn injury recovery. Pediatric burns remain a considerable concern, particularly among males and in household environments. The data underline the imperative for prevention strategies and optimized emergency care to positively influence outcomes for burn victims. Future research areas range from evaluating pre-treatment decisions to assessing community awareness regarding burn first aid.

## Introduction and background

Pediatric burns represent a significant global health concern, accounting for a notable portion of nonfatal injuries among children. The World Health Organization (WHO) estimates that over 180,000 deaths annually result from burns, with the majority occurring in low- and middle-income countries [1]. Pediatric populations are especially vulnerable, given their natural curiosity, limited understanding of risk, and physical environment, often predisposing them to burn injuries. Within this demographic scenario, children aged 0 to 4 years frequently bear the highest risk, predominantly due to scalds and contact burns [2]. Factors contributing to these injuries span from individual behaviors and household environments to broader societal and regulatory elements. For instance, household scenarios, especially those involving food preparation or open flames, have been recurrently pinpointed as substantial risk hubs [3]. Meanwhile, certain cultural practices, lack of public awareness, and limited access to safety equipment further amplify the risks [4]. Moreover, the aftermath of pediatric burns is not merely physical. Burn injuries, particularly severe ones, usher in an array of psychological, social, and economic repercussions. Children might grapple with post-traumatic stress, reduced quality of life, and prolonged hospitalizations, while families often face emotional distress and financial burdens [5]. Thus, understanding the epidemiology, etiology, and outcomes of pediatric burns is paramount to devising effective preventive strategies and optimizing care. Efforts towards prevention and care optimization hinge on comprehensive research. Scrutinizing the etiological factors, gauging the efficacy of prevention programs, and examining treatment regimens and long-term outcomes can furnish invaluable insights. Such rigorous analyses can then pave the way for evidence-based interventions, tailored education campaigns, and informed policy decisions that collectively aim to curb the incidence and improve outcomes of pediatric burn injuries.

## Review

Methods

Study Objective

The primary objective of this systematic review was to explore the epidemiology, outcomes, and management of pediatric burns among patients presenting to emergency departments globally.

Search Strategy

We conducted a comprehensive literature search across multiple electronic databases, including Web of Science, Wiley Online Library, PubMed, Ovid, Google Scholar, and EBSCO. from their inception to December 2022. The following search string was employed to maximize the retrieval of relevant studies: (("pediatric" OR "paediatric" OR "child" OR "children" OR "adolescent" OR "neonate" OR "infant") AND ("burn" OR "burns" OR "scald" OR "scalds" OR "thermal injury" OR "thermal injuries") AND ("emergency department" OR "emergency room" OR "emergency care" OR "accident and emergency") AND ("epidemiology" OR "incidence" OR "prevalence" OR "rate" OR "rates") AND ("outcome" OR "outcomes" OR "prognosis" OR "morbidity" OR "mortality" OR "complication" OR "complications") AND ("management" OR "treatment" OR "intervention" OR "therapy" OR "care")). Manual searches of the references of included articles were also undertaken to identify potential additional studies not captured by the electronic search.

Eligibility Criteria

Studies were considered eligible if they (1) reported on pediatric populations aged 0-18 years; (2) investigated burn injuries, including all types and causes; (3) detailed patient presentation to emergency departments; and (4) discussed epidemiological data, outcomes, complications, or management strategies.

We excluded studies not published in English to ensure a clear understanding and analysis of the data. Opinion pieces were omitted as they do not provide comprehensive data and are often based on individual or subjective viewpoints, which may not be generalizable or relevant to this systematic review. Studies lacking pertinent data were also excluded as incomplete information could compromise the integrity and reliability of our review findings. Further, we excluded studies that reported a meta-analysis or systematic review, economic analysis, animal study, cadaver study, narrative review, or editorial report, as these do not offer primary, patient-specific data relevant to pediatric burns. Studies that did not report on the outcomes of interest, or included patients with injuries other than burns were also left out to ensure the review maintained a focused and relevant approach to pediatric burn injuries.

Data Extraction

Six independent reviewers performed data extraction using a standardized form. Discrepancies were resolved through discussion or, if necessary, a third reviewer. Extracted data included study design, location, population demographics, burn etiology, outcomes, complications, and management strategies (Table 1, 2).

**Table 1:** Characteristics of the included studies

Author(s)	Study design	Country/Location	Population	Sample size (N)	Age (years), SD	Sex distribution
Rawlins et al., 2007 [6]	Prospective Study	UK (Bradford)	Children 0-16 years with burns at ED	208	5 years, SD: N/A	B: 65%, G: 35%
Wibbenmeyer et al., 2003 [7]	Retrospective Study	USA (Iowa)	Children 0-4 years; Young adults 16-24 years	1382	0-24 years, SD: 18.6	B: 841, G: 539 (Missing: 2)
Abramowicz et al., 2019 [8]	Retrospective Study	USA	Patients <18 years with burn injuries at ED	746,593	6 years, SD: N/A	B: 56%, G: 44%
Ewings et al., 2008 [9]	Retrospective Study	USA	N/A	269	N/A, SD: N/A	B: 53%, G: 43%
Tourtier et al., 2010 [10]	Postal Questionnaire	France	Children with burns (2005)	3,258	N/A, SD: N/A	N/A
Cowan et al., 2013 [11]	Retrospective Study	USA	Patients with specific burn diagnoses from Jan 1999-Jan 2009	75	2 months - 18 years, SD: N/A	B: 66.7%, G: 33.3%
Wasiak et al., 2009 [12]	Retrospective Study	Australia	Resident population of Victoria	36,608	0-70+ years, SD: N/A	N/A
Saritas et al., 2013 [13]	Retrospective Study	Turkey	Children ≤18 years with acute burn injury at hospital	2,269	4.85 years, SD: 4.66 (Range: 1 month - 18 years)	B: 1338 (59%), G: 931 (41%)
Glatstein et al., 2013 [14]	Retrospective Study	Canada	Patients <19 years	36	2.5-19 years, SD: N/A	B: 26, G: 10
Kirsch et al., 1996 [15]	Prospective Study	Trinidad and Tobago	Patients <20 years	714	<4, 4-9, 10-14, 15-19 years, SD: N/A	B: 62.6%, G: 37.1%
Othman et al., 2020 [16]	Retrospective Cohort Study	USA	Patients ≤18 years	2,599	Infants (0-1), Toddlers (2-3), Pre-school (4-5), School-age (6-12), Adolescents (13-18), SD: N/A	B: 57.3%, G: N/A
Johnson et al., 2016 [17]	Prospective Study	USA	Children <18 years (using ICD-9 codes 940–949 for burns)	28,363	N/A, SD: N/A	B: 55% (69,728), G: 45% (57,007)
Al-Hoqail et al., 2011 [18]	Prospective Study	Saudi Arabia (Assuming KSA stands for Kingdom of Saudi Arabia)	Population includes children up to 18 years and adults	238	N/A, SD: N/A	B: 59.2% (141), G: 40.8% (97)
Yamamoto et al., 1992 [19]	Retrospective Cohort Study	USA	N/A	18,546	N/A, SD: N/A	B: 9240, G: 9306
Hutchings et al., 2010 [20]	Prospective Case Series	UK	Children <3 years	145	Median: 1.3 years (Range: 0-36 months), SD: N/A	B: 57.2% (83), G: 42.8% (62)
McCormack et al., 2003 [21]	Retrospective Study	Australia	Children with TBSA < 10% (undefined age for children)	109	N/A, SD: N/A	N/A
Battle et al., 2016 [22]	Retrospective Study	UK	Children <16 years	1,387	Median: 2 years (IQR: 1-8), SD: N/A	B: 57.8% (802), G: 42.2% (585)
Yilmaz et al., 2015 [23]	Retrospective Study	Turkey	Children with electrical burns (undefined age for children)	36	6.6 years, SD: 4.71 (Range: 9 months - 15 years)	B: 83.3% (30), G: 16.7% (6)
Choi et al., 2023 [24]	Retrospective Study	South Korea (Assuming you're referring to the Republic of Korea)	Patients <14 years (visited PEDs in South Korea from Jan 1, 2017-Dec 31, 2020)	237,359	<14 years, SD: N/A	N/A
Guzel et al., 2009 [25]	Retrospective Study	Turkey	Patients with scald (at PEU between April 2003-Dec 2007)	165	0-14 years, SD: N/A	B: 57.5% (95), G: 42.5% (70)
Creamer et al., 2008 [26]	Prospective Study	Iraq/Afghanistan	Children 0-17 years	204	9.6 years, SD: 4.9	N/A
McCulloh et al., 2018 [27]	Prospective Study	USA (Boston)	Patients ≤18 years with TBSA ≥10% (presented within 24h of injury at ED)	120	5.4 years, SD: 4.8	N/A

**Table 2:** Management and outcomes of the included studies

Author(s)	Management	Outcome
Rawlins et al., 2007 [6]	Initial Emergency Department (ED) Response: Based on burn severity, patients were either discharged with follow-up, admitted to local plastic surgery, or transferred to a regional burn center. Documentation: All patients had a burn registry form completed in the ED. Dressing: Most burns were treated with liquid paraffin dressings (Jelonet). Referrals: Minor burns were sent to GPs; larger burns were managed in the ED clinic or referred to plastic surgery. Guidelines: The British Burn Association (BBA) guidelines determined referrals, considering burn size, location, and type. Follow-up: Missed appointments led to outreach through letters or home visits by nurses for care and education.	Demographics: 208 children attended the ED, mostly infants and young children. Burn Types: 51% scalds, 36% contact burns. Pre-ED Care: One-third lacked first aid; 87% received no analgesia. Post-ED Directives: 5% discharged with no follow-up. 23% referred to general practitioners. 58% to ED clinic for review. 4% to plastic surgery dressing clinic. 7% admitted to plastic surgery. 3% transferred to a burn center. Surgical Care: 3% needed burn excision and skin grafting. Mortality: No deaths reported.
Ewings et al., 2008 [9]	Many minor injuries, however, are treated in EDs or outpatient settings. The study was a retrospective review conducted over a 5-year period in an urban children's hospital ED to evaluate the management of pediatric upper extremity burns. The aim was to determine the effectiveness of treatments and interventions, especially given the large number of burn patients managed by primary care providers.	75% of the burns were second-degree, 21% first-degree, and 2% third-degree. 15% (40 patients) had a consultation with plastic surgery, and 3% (7 patients) required skin grafting.
Tourtier et al., 2010 [10]	The focus was also on pain management, with 65% of EDs having a written protocol for managing pain in children. For analgesia, 80% of EDs used oxygen/nitrous oxide. Concerning second-step analgesics, 67% used a combination of paracetamol/codeine, while only 22% used non-steroidal anti-inflammatory drugs (NSAIDs). For third-step analgesics, 67% of EDs used nalbuphine, while only 43% used morphine.	These EDs treated a total of 3,258 children with burns, representing 0.63% of pediatric pathologies in EDs.
Saritas et al., 2013 [13]	The patients were managed in accordance with the guidelines of the American Burn Association (1990). The study is retrospective and covers medical records of children (aged 18 years and below) with acute burn injuries admitted to the hospital from January 1, 2001, to December 31, 2008. They were categorized based on various factors like age, cause of the burn, anatomical areas affected, and depth of the burn, among others. The outcomes were classified as either survivor or died.	A total of 2269 children with acute burn injuries were admitted. Out of these, 86 children (3.8%) died due to burn injuries. Deaths were seen 1.849 times more in males than in females. In terms of TBSA (Total Body Surface Area) burned, mortality occurred 121.116 times more in the >41% TBSA burned group. Most deaths (n = 77) were among patients referred to the hospital. The mortality rate was higher in rural areas (6.3%) compared to urban areas (2.8%). Deaths were most frequent in patients with scalding burns, but the mortality ratio was higher for tandir burns. Most of the deaths occurred within the first 10 days of hospitalization.
Glatstein et al., 2013 [14]	28 patients were treated conservatively with dressings and minor surgical interventions like debridement and primary repair. The remaining patients required more extensive treatments like excision and/or grafting. No patient needed amputation. 2 patients underwent fasciotomy and/or escharotomy.	13% of patients with electrical current burns required hospital admission. 60% of patients with lightning-associated burns needed hospitalization. Two patients required prolonged hospital stays after sustaining burns from household electrical incidents. One of these patients, a 7-year-old boy, had burns from electric transmission lines in an industrial area, resulting in third-degree burns to the shoulder, upper limb, trunk, hip, and lower limb, covering approximately 25% of his body surface area.
Al-Hoqail et al., 2011 [18]	Not operated: 168 cases (71.8%) Selection: 71.8% of patients weren't operated due to reasons like minor burns (78.5%), patient refusal (1.8%), transfers (1.8%), or high anesthetic risk (0.6%). Wound Care: Clean and dress burns regularly. Pain Management: Administer pain relief as needed. Monitoring: Check for healing progress and signs of infection. Rehabilitation: Physical therapy for affected areas if necessary. Education: Advice on home care and potential complications. Operated: 66 cases (28.2%) Most common first operation: Split-thickness skin grafting surgical interventions can range from debridement to skin grafting. The urgency of operations was categorized as: Elective (60.6%): These operations are scheduled in advance and aren't emergencies; Emergency (39.4%): Immediate surgical intervention is required.	Burns that were surgically excised and grafted between 12 and 18 days recovered more quickly than those managed conservatively until the eschar had sloughed off, followed by grafting. Burns managed by excision and grafting in less than 5 weeks post-injury healed 8.6 days faster than those grafted later. For more severe burns, those grafted within 5 weeks healed 13 days faster than the delayed group.
McCormack et al., 2003 [21]	Immediate Cooling: Run cold tap water directly onto the burn for a minimum of 20 minutes. Be cautious to prevent hypothermia. Maintain Patient Warmth: Elevate the room's temperature to 25°C–30°C. Remove any wet clothing from the patient and cover unburnt areas with a blanket. Continuous Cooling During Transport: Use a fine mist spray or frequently changed soaked dressings to continue cooling the burn while transporting the patient. Avoid Ice: Never apply ice to a burn. Timeliness: Administering first aid within the first three hours post-burn is beneficial.	14 out of 14 (100%) had presented to their general practitioner (GP). 22 out of 31 (71%) had presented to their local hospital. 22 out of 38 (58%) had presented to CHW. 2 out of 2 (100%) had first contact with other health professionals.
Yilmaz et al., 2015 [23]	Urine Output Monitoring: Patients had their urine output monitored. Electrocardiography (ECG): ECG findings were recorded for each patient. Serum Measurements: Serum values for alanine transaminase (ALT), aspartate transaminase (AST), creatine kinase (CK), and creatine kinase-myocardial isoenzyme (CK-MB) were recorded. Fluid Resuscitation: All patients underwent fluid resuscitation. Burn and Wound Care: Burn and wound dressings were applied to the patients. Tetanus Prophylaxis: Tetanus prophylaxis was provided as indicated.	Mortality: There were no reported deaths among the patients. Recovery: The specific recovery details or long-term outcomes for the patients were not provided in the paper

Quality Assessment

The studies' quality was examined using the methodological index for non-randomized studies (MINORS). MINORS is an approved instrument for gauging the methodological integrity of non-randomized research types such as cohort, case-control, and comparative observational studies. For non-comparative studies, it has 12 criteria, and comparative ones, it has 8, with each criterion rated between 0 and 2. Non-comparative studies can achieve up to 16 points, while comparative ones can reach 24 points. The quality of the selected studies was independently reviewed by two authors using the MINORS, and any disputes were settled via dialogue or by seeking the opinion of a third author [28].

Data Analysis

Descriptive statistics were employed to summarize the extracted data. Depending on the heterogeneity of the included studies, a meta-analysis would be considered using random-effects models. Pooled estimates were calculated for key outcomes, such as burn incidence rates, morbidity, mortality, and complication rates.

Reporting

**Figure 1:**
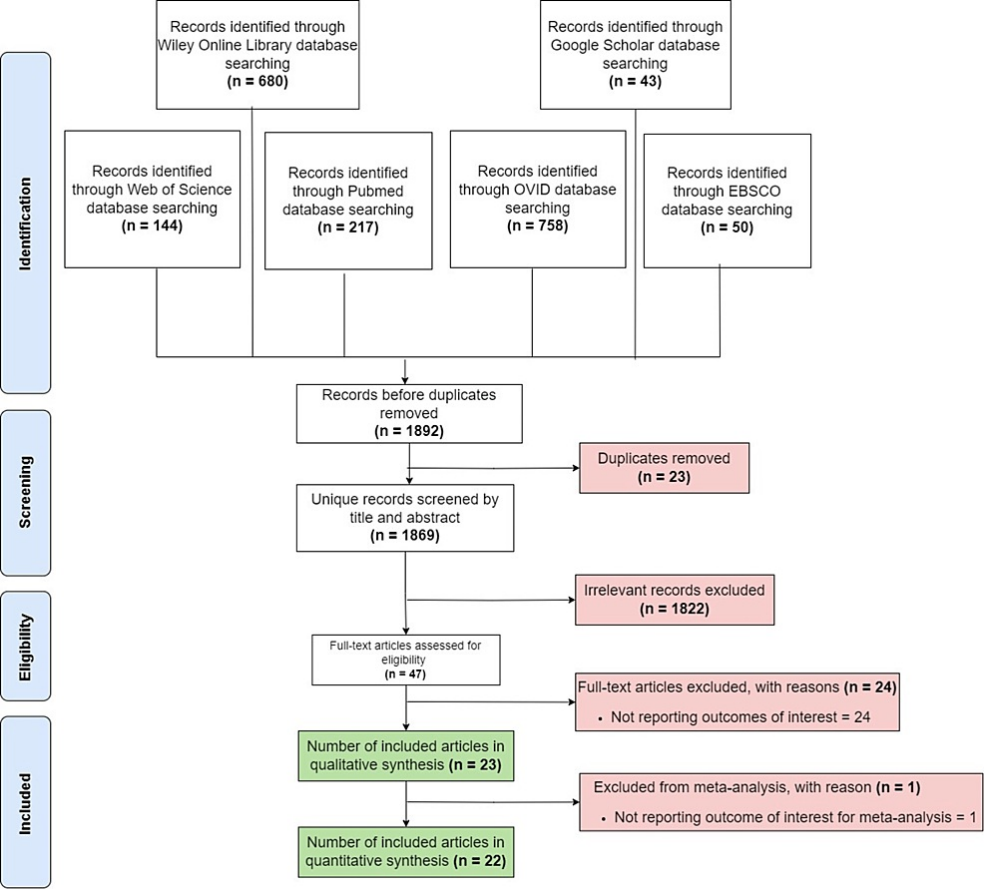
PRISMA flowchart, representing the full process of article inclusion PRISMA, Preferred Reporting Items for Systematic Reviews and Meta-Analyses

This review adheres to the Preferred Reporting Items for Systematic Reviews and Meta-Analyses (PRISMA) guidelines to ensure comprehensive and transparent reporting (Figure 1).

Results

MINORS Evaluation

For this review, the MINORS instrument was employed to gauge the quality of the non-randomized studies. The cumulative scores for non-comparative studies spanned from 2 to 11, averaging 6.87, while for comparative studies the scores ranged from 12 to 16, averaging 13.67. Criteria that consistently scored low included the forward-looking calculation of study size (every study scored 0), objective assessment of the study endpoint (each study had a score of 0), and the sequential inclusion of patients (most studies scored either 1 or 2). On the other hand, aspects that achieved top scores were the explicitly expressed study objective, detailed patient demographics, and well-defined endpoints, all of which scored 2 in every study (Tables 3, 4; Appendix A; Appendix B).

**Table 3:** Risk of bias (comparative studies)

Authors	A clearly stated aim	Inclusion of consecutive patients	Prospective collection of data	Endpoints appropriate to the aim of the study	Unbiased assessment of the study endpoint	Follow-up period appropriate to the aim of the study	Loss to follow up less than 5%	Prospective calculation of the study size	An adequate control group	Contemporary groups	Baseline equivalence of groups	Adequate statistical analyses	Score out of 24
Aslıhan Arasli Yilmaz et al., 2015 [23]	2	1	0	2	2	0	0	0	2	2	0	1	12
Arum Choi et al., 2023 [24]	2	0	0	2	2	2	0	0	2	2	2	2	16
Christopher McCulloh et al., 2018 [27]	2	1	0	2	2	0	N/A	0	2	1	0	2	12
Sarah A. Johnson et al., 2016 [17]	2	1	0	2	2	0	N/A	0	1	2	0	2	12
Rola Abdullah Al-Hoqail et al., 2011 [18]	2	2	2	2	2	N/A	N/A	2	1	1	0	1	15
H. Hutchings et al., 2010 [20]	2	2	0	1	2	1	2	N/A	2	1	0	2	15

**Table 4:** Risk of bias (noncomparative studies)

Authors	A clearly stated aim	Inclusion of consecutive patients	Prospective collection of data	Endpoints appropriate to the aim of the study	Unbiased assessment of the study endpoint	Follow-up period appropriate to the aim of the study	Loss to follow up less than 5%	Prospective calculation of the study size	Score out of 16
Jeremy M. Rawlins et al., 2007 [6]	2	2	1	0	0	2	0	0	7
Lucy Ann Wibbenmeyer et al., 2003 [7]	2	1	1	0	0	0	0	2	6
Shelly Abramowicz et al., 2019 [8]	2	2	2	0	0	1	2	2	11
Ember Lee Ewings et al., 2008 [9]	2	2	0	0	0	2	0	0	6
J. P. Tourtier et al., 2010 [10]	2	2	2	0	0	0	0	1	7
Douglas Cowan et al., 2013 [11]	2	2	2	0	0	1	N/A	0	9
Jason Wasiak et al., 2009 [12]	2	N/A	0	0	0	N/A	N/A	0	2
Ayhan Saritas et al., 2013 [13]	2	1	2	0	0	N/A	N/A	0	5
Miguel M. Glatstein et al., 2013 [14]	2	2	2	0	0	N/A	N/A	2	8
Kevin M. Creamer et al., 2008 [26]	2	1	0	2	2	0	N/A	0	7
Ahmet Guzel et al., 2009 [25]	2	0	0	2	2	0	N/A	0	6
Ceri Elisabeth Battle et al., 2016 [22]	2	1	0	2	0	N/A	N/A	0	5
Thomas D. Kirsch et al., 1996 [15]	2	2	0	1	0	0	N/A	N/A	5
Sammy Othman et al., 2020 [16]	2	2	0	2	2	N/A	N/A	2	10
L. G. Yamamoto et al., 1992 [19]	2	1	2	0	0	2	N/A	1	8
Rebecca A. McCormack et al., 2003 [21]	2	2	2	1	0	N/A	N/A	N/A	7

*Study Design and Location * 

A total of 22 studies were identified for inclusion in the systematic review. The studies were conducted between 1992 and 2023. Most studies utilized a retrospective design, with a few employing prospective methods. The geographic distribution of the studies spanned multiple continents, including North America, Europe, Asia, and Australia.

Study Populations and Sample Sizes

The total combined sample size of all included studies was 828,538 pediatric patients. The youngest age group investigated was children aged 0 to 4 years, while the eldest group ranged up to 18 years. Several studies, such as those by Wibbenmeyer et al. and Saritas et al. narrowed their focus on specific age groups, while others, like that by Wasiak et al. encompassed a broader age range [7,12,13].

Sex Distribution

Across the studies, there was a noticeable male predominance in pediatric burn victims. For instance, Rawlins et al. reported 65% boys and 35% girls, while Abramowicz et al. observed 56% males and 44% females [6,8]. Similar trends were seen in other studies, reinforcing the observation that boys might be at a higher risk for burn injuries.

**Figure 2:**
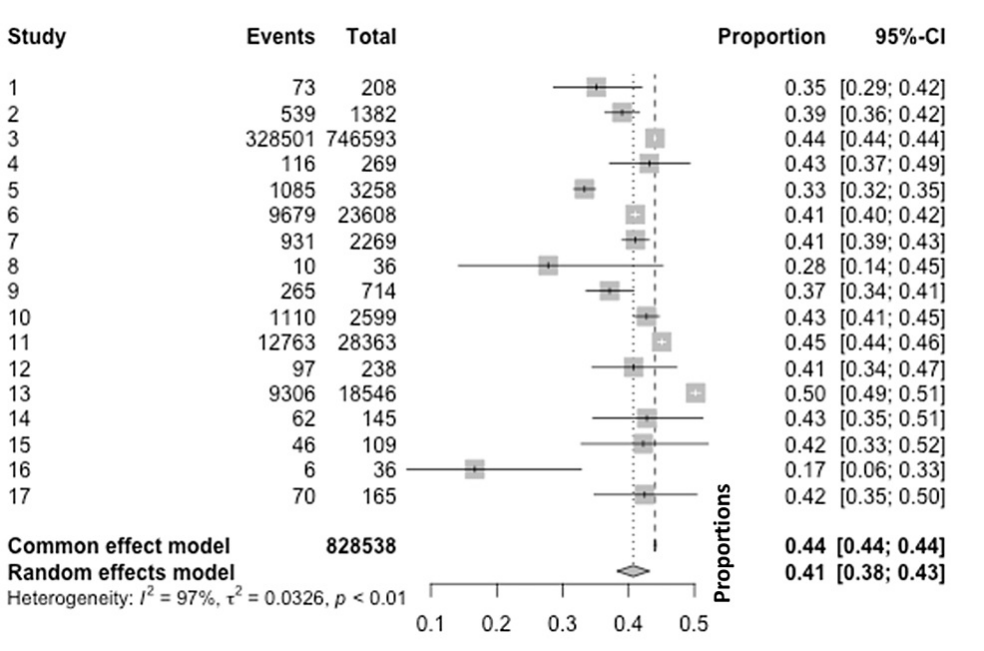
Meta-analytic overview of burn incidence in girls

Girls' meta-analysis: The meta-analysis for girls covered various studies that investigated the proportion of burn injuries among girls. A total of 828,538 events were observed across these studies. Using a common effect model, a considerable variation in burn proportions among girls was observed across the different studies, ranging from 17% to 50%. When applying a random-effects model to account for potential heterogeneity among the studies, substantial heterogeneity was detected with I2=97I2=97, tau (ττ) of 0.0326, and a statistically significant p-value of <0.01. This high I2I2 value suggests that 97% of the observed variance reflects real differences in effect sizes, rather than sampling error (Figure 2).

**Figure 3:**
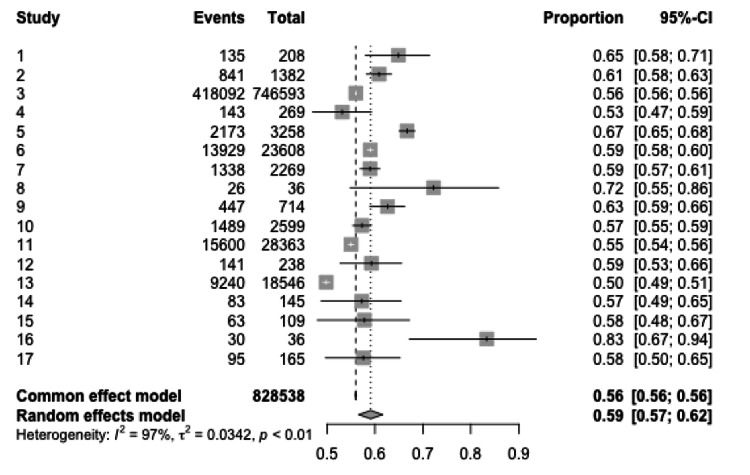
Meta-analytic overview of burn incidence in boys

Boys’ meta-analysis: The meta-analysis for boys also covered various studies, looking at the proportion of burn injuries among boys. A total of 828,538 events were observed, mirroring the total for girls, indicating potentially similar datasets. With a common effect model, the observed proportions of burns in boys varied from study to study, with values ranging from 53% to 83%. In the random-effects model, considerable heterogeneity among the studies was evident with an I2I2 value of 97%, a tau (ττ) of 0.0342, and a p-value of <0.01, suggesting that the observed variability in burn proportions is real and not just due to sampling variability (Figure 3).

Etiology of Burns

**Figure 4:**
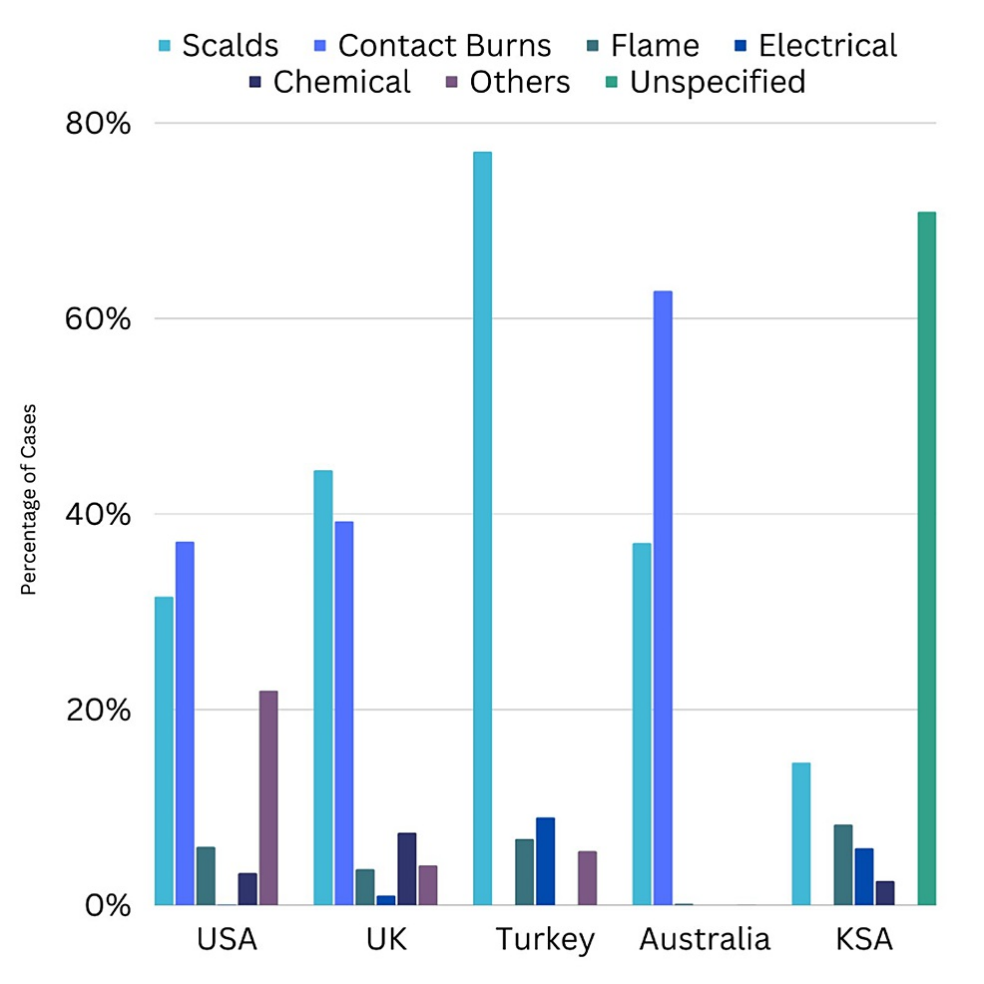
Types of burn injuries by geographic location

Scalds emerged as a prominent cause of pediatric burn injuries, with numerous studies, including those by Rawlins et al. and Yilmaz et al., reporting them as the leading etiology [6,23]. Contact burns, electrical burns, flame-related injuries, and other specific causes such as fireworks or chemical agents were also identified. The types of burn injuries by geographic location are shown in Figure 4.

Burn Degree and Total Body Surface Area (TBSA)

The studies revealed variability in burn degrees. Rawlins et al. report that a majority (89%) of the injuries were second-degree burns, while Ewings et al. found that 75% of patients had second-degree burns [6,9]. The data on TBSA was inconsistent among studies. Some studies, like the one by Ewings et al., provided detailed breakdowns of TBSA percentages, while others, such as the study by Wibbenmeyer, et al. did not offer specifics [7,9].

Identified Risk Factors

Several studies identified specific risk factors for pediatric burn injuries. Rawlins et al. pointed out a male predominance, reflecting higher risk activity among older boys [6]. Cowan et al. highlighted the dangers associated with heating formula prior to feeding, and the ingestion of hot food, liquids, hair products, and household cleaners [11]. Moreover, the risk from heating liquids over a stove, oropharyngeal burns from hair products, and the potential for ingestion of batteries or cleaning detergents were noted. Wasiak et al. observed that males, children aged less than five years, and the elderly were at the highest risk [12]. In addition, contact with heat and hot substances was a significant contributor to thermal injuries. Saritas et al. emphasized a higher mortality ratio in rural patients, attributing it to lower educational and socioeconomic levels, and highlighted the increased vulnerability of children under five [13]. Hutchings noted home-based risks such as proximity to ironing and incidents during mealtimes [20]. Battle et al. recognized commonalities in burn occurrences, mainly in 1-2-year-olds due to hot liquids and household devices [22]. Yilmaz et al. found electric shocks were more common in males across all age groups [23].

In the risk factors associated with pediatric burns, a distinct pattern emerges, highlighting several critical areas of concern. Male children, particularly those under five years of age, exhibit a heightened vulnerability to burns, underlining a crucial need for targeted preventive measures within this demographic. The domestic environment emerges as a significant arena for potential hazards, with activities such as heating formulas and the ingestion of various household substances presenting notable risks. Rural settings further exacerbate these risks, with a demonstrated increase in mortality ratios, pointing to the impact of educational and socioeconomic factors on burn incidence and outcomes. The data further identify specific types of burns, with electrical burns being more prevalent in males across all age groups, underscoring the necessity for focused educational interventions and safety measures. The encapsulation of these risk factors provides a crucial blueprint for future preventive strategies, emphasizing the importance of a multifaceted approach to risk reduction in pediatric burn incidents.

Interventions and Management

Ewings described interventions like immediate resuscitation, fasciotomy, and skin grafting [9]. Cowan et al. provided a detailed management strategy where systemic steroids were used in 8% of admitted cases, 30% received antibiotic coverage (with clindamycin being the most common), and 33.3% of patients were made NPO [11]. Various surgical interventions, including debridement, escharotomy, and skin grafting, were mentioned across the studies. McCormack et al. recommended cooling by tap water for over 20 minutes as a primary intervention, while Yilmaz et al. stressed the importance of initial ECG testing for electric shock cases [21,23].

Immediate and Short-Term Outcomes

Rawlins et al.'s data reflected that 5% of patients were discharged immediately, with a significant portion being instructed to return to the emergency department [6]. Wibbenmeyer et al. reported that most patients were treated in the emergency department and then sent home, with only 2.5% requiring hospitalization [7]. Post-treatment, patients had the option to see their primary physician, return to the emergency department, or consult with a plastic surgeon. The study by Ewings and Pollack documented functional outcomes for 5% of patients, with varied results, whereas Cowan et al. provided a detailed account of treatments such as systemic steroids, antibiotics, and surgical interventions [9,11]. Othman et al.'s findings showed that the majority of patients were treated and released directly from the emergency department [16].

Long-Term Outcomes

A few studies discussed long-term outcomes. For instance, Wasiak et al. evaluated the data of 34,343 patients treated in hospitals for non-fatal burns over a span of seven years [12]. Clinical recommendations and expected long-term outcomes associated with electrical shock burns are detailed in Table 2.

Potential Areas for Future Research 

Pre-treatment decisions: Research is needed to understand why some pediatric burn patients do not receive initial first aid or analgesia prior to emergency care, pinpointing public knowledge gaps or access barriers.

Comparison of care: Outcomes between emergency departments and specialized burn centers must be compared to determine the most effective treatment settings.

Follow-up care: An exploration into the varied follow-up care options and their effectiveness is essential for improving long-term outcomes in pediatric burns.

Burn prevention: The efficacy and outreach of burn prevention programs should be rigorously assessed to refine and augment them.

Household risks: In-depth research on household burn hazards, especially from everyday items and meal preparation, can illuminate prevention areas.

Regulatory impacts: Changes in pyrotechnics regulations and their influence on burn injuries, especially among children, warrant study to guide safer policies.

Treatment protocols: Research on prescribed treatments, patient adherence, and outcomes can offer insights into best practices for pediatric burn care.

Digital advancements: Leveraging evolving electronic medical records can lead to richer, more comprehensive data collection, aiding in-depth analysis.

Referral evaluation: Assessing the current American Burn Association (ABA) referral criteria for pediatric burns can ensure optimal patient care routing.

Quality management: Implementing total quality management-burn injuries (TQM-BI) may identify and bridge care gaps.

Centralized data: A unified data collection system can boost research accuracy and consistency in documenting burn injuries.

Socio-environmental Impacts: Investigating the role of family dynamics, frequent relocations, and social support in burn injury recovery can offer a holistic perspective.

Educational outcomes: Analyzing the educational paths of burn survivors can provide valuable insights into necessary academic support systems.

Community awareness: Assessing and enhancing community knowledge on burn first aid is crucial for early intervention.

Prevention effectiveness: Evaluating the actual impact of current burn prevention strategies will inform future public health initiatives.

Discussion

Pediatric burns, a significant global health concern, often lead to severe morbidity, considerable health care expenses, and profound psychosocial implications. This systematic review aimed to provide an in-depth understanding of the epidemiology, outcomes, and management of pediatric burns presenting to emergency departments.

The predominance of male pediatric patients with burn injuries aligns with earlier studies, suggesting that boys may inherently engage in more risk-taking behaviors or may have different exposure to burn risk factors than girls [3,4,29]. Further investigation into these behavioral and environmental factors is crucial to tailor prevention programs effectively.

Interestingly, our review identified scalds as a leading cause of burn injuries, consistent with other literature indicating that hot liquids, steam, or foods are primary hazards for younger children [29]. This finding underscores the importance of interventions focusing on household hazards and parental education to reduce the incidence of these preventable injuries.

The observed gender disparity in pediatric burns, where males are more frequently victims, may stem from various reasons. One possible explanation could be the societal and cultural expectations that shape children's behaviors from a young age. Boys are often encouraged to be adventurous and engage in explorative and sometimes risk-associated activities more than girls, leading to a higher likelihood of exposure to situations that can result in burns. In addition to societal influences, biological factors might also play a role. Boys generally have higher impulsivity and reduced risk perception compared to girls, making them more prone to accidents, including burns.

The differences in activities and toys that are traditionally assigned to boys may also contribute to this increased risk. For instance, boys may be more likely to be involved in activities or play with toys that have a higher association with fire or heat, such as playing in outdoor settings where they might be exposed to open flames. The observed male predominance in pediatric burn victims is a significant finding, warranting an in-depth exploration to understand the underlying behavioral and situational factors. The male inclination for high-risk activities may play a role in the increased incidence of burns, necessitating targeted prevention and intervention strategies.

The variations observed in burns among girls and boys, as evidenced in the meta-analysis, further highlight the need for gender-specific approaches to both prevention and treatment. The variability in burn degrees and TBSA percentages across studies may be attributed to diverse data collection methods and geographical variations in burn causes and mechanisms. Notably, studies like those by Ewings and Pollack provided insights into the prevalence of second-degree burns [9]. Such data can guide clinicians in developing treatment and management plans specific to the burn degree.

This review also highlighted household scenarios, particularly around mealtimes, as significant risk factors for pediatric burns. Similar observations have been reported in the past, emphasizing the role of household environments in burn injury occurrences [3,4,29]. The emergency department serves as a pivotal initial point of care for burn victims.

Our findings emphasize the need to equip emergency departments with specialized knowledge and resources to optimize the care of pediatric burn patients. Previous research has highlighted the potential benefits of integrating specialized burn care within emergency department settings for improved patient outcomes [30]. 

Recommendations

Focus on male children: Given the male predominance in pediatric burn victims, targeted educational and awareness campaigns should specifically address boys' heightened risk, potentially influencing behavioral changes to reduce injury incidence.

Scald prevention: Owing to the prominence of scalds as a leading cause of pediatric burn injuries, parents and caregivers need targeted education about household hazards associated with hot liquids and foods. Proper storage and handling of hot substances should be emphasized.

Standardize data collection: Considering the variability in burn degrees and TBSA reported, standardization in data collection across healthcare institutions might allow for a more coherent understanding of burn injury severity.

Household safety: Efforts should be intensified to educate families about burn risks during mealtimes, emphasizing safe practices during cooking and serving hot foods.

Strengthen emergency department protocols: Specialized training and resources should be made available to emergency department staff to ensure optimal initial care for pediatric burn victims, potentially reducing long-term morbidity.

Research expansion: As electronic medical record systems become increasingly integral in healthcare, they should be leveraged to facilitate broader, in-depth research into pediatric burns, aiding in prevention, and treatment strategies. The outlined potential areas for future research are invaluable. With electronic medical record systems becoming commonplace, future studies harnessing these databases can potentially facilitate more extensive and detailed research on pediatric burns. This review elucidates vital aspects of pediatric burns, from their epidemiology and causes to treatment outcomes. The highlighted findings underscore the importance of targeted interventions, community awareness, and continuous research in this domain to further enhance patient care and outcomes.

*Study Limitations* 

Although interesting, this systematic review and meta-analysis on pediatric burns has a number of drawbacks. It might not accurately reflect the needs and experiences of female patients given its large proportion of male patients. The generalizability of the findings may be impacted by regional variations and variations in data collection techniques among the included studies. The review did not delve into the long-term care and rehabilitation aspects of burn management due to its focus on emergency department care. Moreover, a thorough examination of the psychosocial effects of burns has not been conducted, which could lead to the omission of important patient care components. Furthermore, even though they are detailed, the review's recommendations might not cover all required measures. These drawbacks emphasize the necessity of larger, more inclusive studies in order to fully address pediatric burns.

## Conclusions

This comprehensive review of pediatric burns underscores their significant impact on public health, particularly affecting young males predominantly within household settings. The analysis reveals scalds as the primary cause of these injuries, often resulting in second-degree burns. However, the need for hospitalization appears limited, as evidenced by the small percentage of cases requiring such intervention. The findings highlight the necessity of targeted prevention strategies and the refinement of emergency response protocols to improve patient outcomes. Moreover, the review identifies critical gaps in current knowledge, particularly concerning household risk factors, initial treatment decisions, and the influential role of family dynamics in the recovery process. Addressing these areas in future research is essential to enhance community awareness, especially regarding effective first aid for burns, and to develop more effective measures for both prevention and management of pediatric burn injuries.

## Figures and Tables

**Figure 5:**
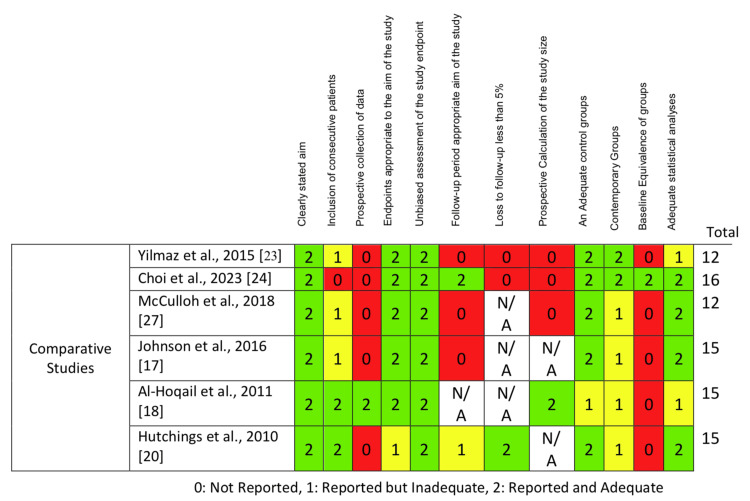
Risk of bias summary figure for comparative studies using the MINORS tool MINORS, methodological index for non-randomized studies

**Figure 6:**
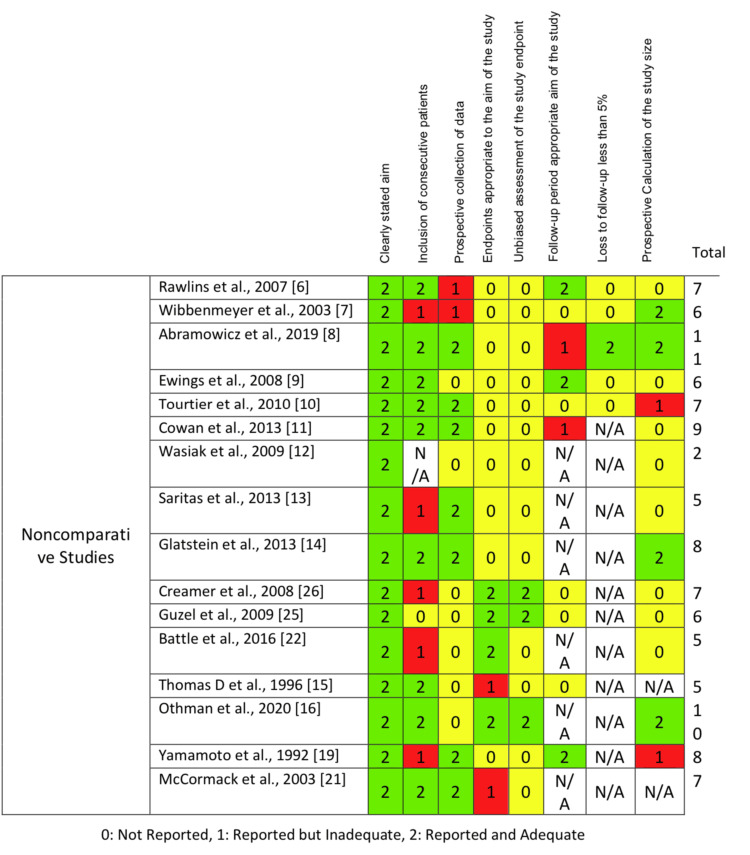
Risk of bias summary figure for noncomparative studies using the MINORS tool MINORS, methodological index for non-randomized studies

## Appendices

Appendix A

 Appendix B
